# Port Placement via the Anterior Jugular Venous System: Case Report, Anatomic Considerations, and Literature Review

**DOI:** 10.1155/2017/2790290

**Published:** 2017-04-10

**Authors:** Gernot Rott, Frieder Boecker

**Affiliations:** Radiological Department, Bethesda-Hospital Duisburg, Heerstr. 219, 47053 Duisburg, Germany

## Abstract

We report on a patient who was referred for port implantation with a two-chamber pacemaker aggregate on the right and total occlusion of the central veins on the left side. Venous access for port implantation was performed via left side puncture of the horizontal segment of the anterior jugular vein system (AJVS) and insertion of the port catheter using a crossover technique from the left to the right venous system via the jugular venous arch (JVA). The clinical significance of the AJVS and the JVA for central venous access and port implantation is emphasised and the corresponding literature is reviewed.

## 1. Introduction

Venous access for chest port implantation may be compromised for a variety of reasons and finding a suitable alternative can be difficult. If a conventional approach for this purpose is not an option, collateral veins may offer an attractive alternative. A crossover route utilising the anterior jugular venous system (AJVS) and the jugular venous arch (JVA) may be considered. We present, to the best of our knowledge, a unique case where anatomical constraints led us to utilise this crossover technique for port placement.

The placement of a chest collateral port in crossover technique through the anterior jugular vein system can be quite simple and the most appropriate method in the mentioned or a comparable scenario.

## 2. Case Report

**Figure 1:**
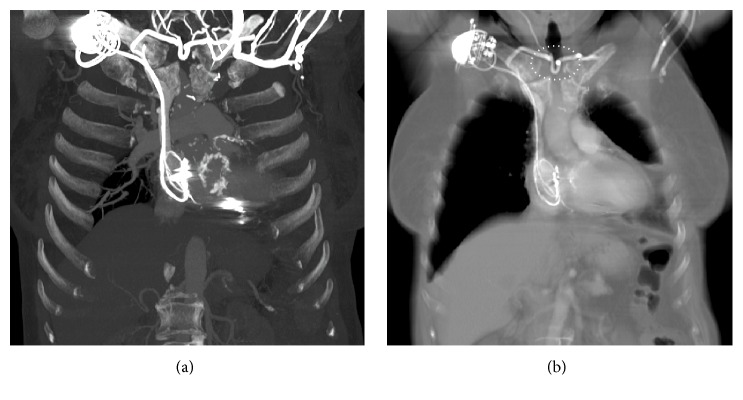
CT with administration of contrast media through a left peripheral vein. (a) Coronal maximum intensity projection image shows chronic occlusion of the left-sided central veins with cervical collateral veins from the left to the right side and here reaching the nonoccluded right-sided central veins; slight metal artifacts by the right-sided pacemaker. (b) Coronal multiplanar reconstruction image showing a partially quite tortuous u-shape horizontal-transverse midline vein in the jugulum (dotted ellipse), the AJVS with the JVA.

A 70-year-old woman was admitted to our hospital for staging and therapy of a recently diagnosed adenocarcinoma of the esophagogastric junction. The significant past medical history of the patient included implantation of a two-chamber pacemaker on the right side and subtotal sternum resection with a pectoral muscular flap graft coverage for treatment of sternum osteomyelitis. Contrast enhanced computed tomography (CT) of the thorax showed chronic occlusion of the left-sided central veins with the presence of a cervical collateral venous flow from the left to the right side reaching the nonoccluded right-sided central veins, specifically the confluence to the right innominate vein (IV) and, as part of the collateral circulation, a transverse connecting, in parts quite tortuous, and u-shape midline jugular vein measuring about 3 mm in diameter (Figure 1). Staging examinations revealed a T3 cancer and our interdisciplinary tumor board recommended the implementation of neoadjuvant chemotherapy.

The patient was presented to our radiological department for port implantation. We decided to opt for venous access on the left upper body to avoid problems with the pacemaker aggregate on the right upper body. The plan was to recanalise the left IV. If this proved unsuccessful, alternative access via puncture of a left-sided collateral vein to reach the contralateral central veins would be instigated.

**Figure 2:**
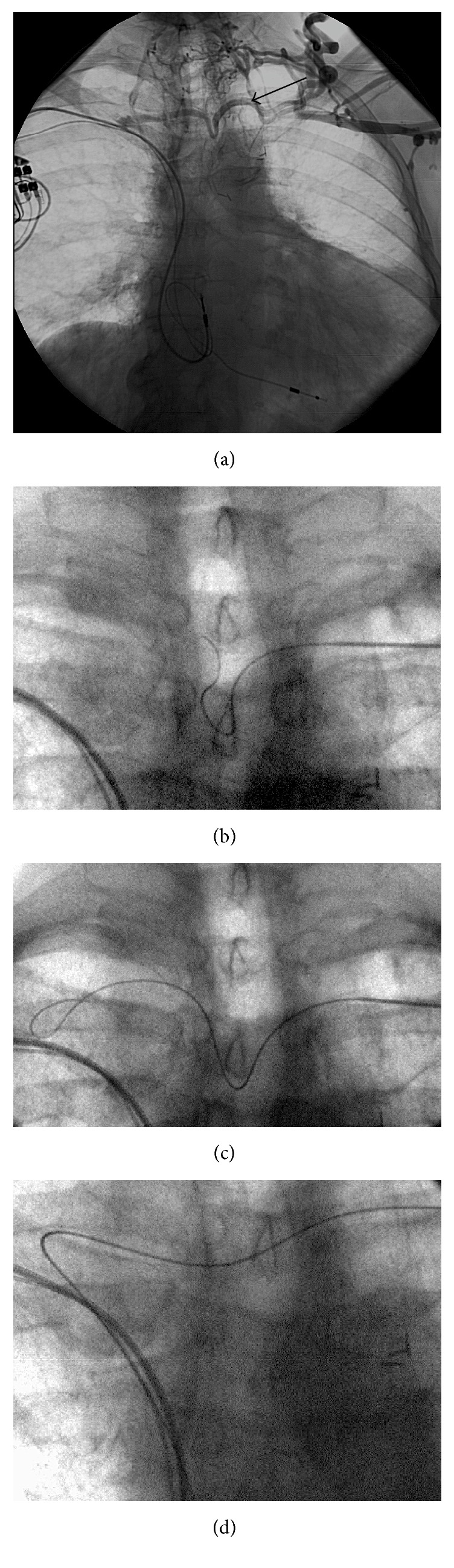
Phlebography correlation of the left arm demonstrates the same collateral circulation as in the CT, fully comparable to Figure 1(a) (a). Note arrow indicating the exact location of the following vascular access. (b–d) Fluoroscopy with last-image hold images showing a guidewire stepwise navigated through the AJVS crossover to the right side and from here through the right innominate vein along the pacemaker leads into the inferior vena cava.

**Figure 3:**
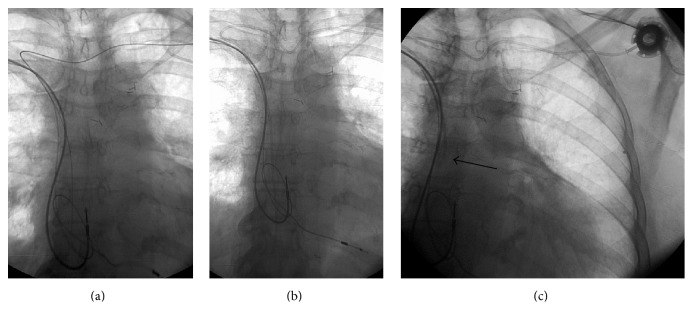
Fluoroscopy-guided images showing guidewire (a) and catheter (b) placement into the inferior vena cava and final positioning of the venous port system (c).

The procedure was performed under local anesthesia in our angiographic suite. Phlebography of the left arm demonstrated the venous anatomy of the shoulder region, as described above in the previously performed CT (Figure 2). After the usual preparations, the left subclavian vein (SV) was punctured under fluoroscopy and guidance of contrast venography, and a 4-French catheter was introduced. Recanalisation of the totally occluded left IV was attempted using different types of guidewires, however without success due to high tissue resistance and absence of a funnel-shaped stump in the area of the chronic occlusion. After another review of the CT series, a left-sided thyrocervical transverse collateral vein, localised relatively superficially and directly above the sternal end of the left clavicle, was selected. This vessel was easily punctured with an 18-gauge needle using solely the bony landmark of the left clavicle. A 0.035-inch angled tip hydrophilic guidewire was stepwise navigated through the tortuous midline u-shaped segment of the collateral vein and crossed over to the right side and from here at an acute angle via the right IV, along the pacemaker leads into the inferior vena cava (Figure 2). The 6-French catheter of the port set (Titanium Low-Profile Implantable Port, Bard Access System Inc., Salt Lake City, USA) was then inserted over the wire just as easily and without complication or kinking up to the cavoatrial junction. A port pocket was prepared below the lateral end of the left clavicle, and the port catheter was tunnelled from the puncture side to the pocket completing the procedure as usual (Figure 3). The port could be used for the entire chemotherapy without any problems.

## 3. Discussion

In our institution, both radiologists and surgeons utilise the anterior chest wall for the standard approach for port placement. As insertion of arm ports does not necessarily show always the best results [1, 2], we consider peripherally placed central venous access ports only to be an alternative for patients in whom a chest-placed device is inappropriate or undesired.

For patients with implantable cardiac devices, the general recommendation is to place the port on the contralateral side to avoid any damage to pacing or defibrillator leads during port placement [3, 4]. Furthermore, ipsilateral central venous access along with a pacemaker is a compounding risk variable for central venous occlusion. Although ports are much smaller and less bulky than a pacemaker, positioning a port chamber directly next to a pacemaker aggregate or pacemaker lead in our experience is not a good solution. In such a situation, the options for port-chamber placement are clearly limited and the port chamber may cause an uncomfortable result for the patient due to its proximity to the clavicle.

In our patient, we considered a right-sided approach via the internal jugular vein (IJV) with tunnelling the catheter over a long distance to the other side, but we refrained from this because of the extended presternal scars after sternal osteomyelitis and subtotal sternal resection.

In this clinical case, we proposed port placement at the left upper body to avoid problems with the pacemaker aggregate on the right side.

Translumbar port placement had been discussed with the patient as an alternative, in the event of failed thoracic placement.

## 4. Anatomical Considerations

A closer look at the anatomical conditions of our patient reveals that the supraclavicular punctured collateral vein segment is the horizontal lateral segment of the AJVS, as has been described and defined under functional and clinical aspects by Chasen and Charnsangavej [5] and Schummer et al. [6]. The midline u-shape thyrocervical collateral vein in fact is the JVA (Terminologia Anatomica: arcus venosus jugularis).

The JVA is an infrequently found transverse connecting trunk extending across the midline between the two anterior jugular veins (AJV) of either side and lying in the suprasternal space between superficial and pretracheal layers of the cervical fascia. The JVA serves as a natural crossover collateral and may become prominent in cases of deep venous outflow obstruction. It is the midline part of the AJVS, typically in u-shape or v-shape configuration. Apart from textbooks of surgery in the context of, for example, thyroid surgery or tracheostomy, it is mentioned in the literature mostly in relation to malposition of central vein catheters or unintended crossover placement of central lines [7, 8].

The AJVS is an important collateral venous network across the midline of the superoanterior aspect of the thorax and, if fully developed, is composed of three segments: the JVA as the transverse midline segment [5] and the two as infrequently found horizontal lateral segments connecting the JVA to the subclavian vein (SV), external jugular vein (EJV), or more rarely IJV. It is worth emphasising that “anterior jugular venous system” is to be understood as a clinical and not as an anatomical term. In the anatomical images or the textbooks of anatomy, the horizontal lateral segment of the AJVS often is unmarked or rather regarded as the termination of the AJV. Corresponding to its variability, the AJVS shows a wide array of formation, course, communication, and termination [9].

**Figure 4:**
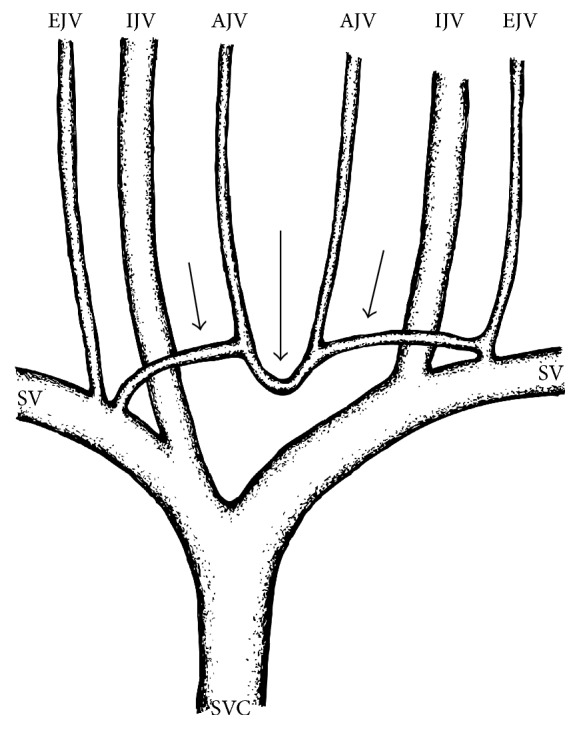
Schematic drawing illustrating the segmental anatomy of a typically and fully developed anterior jugular venous system (AJVS) consisting of three segments (three arrows) with the jugular venous arch (JVA) as the transverse midline segment (large arrow). AJV: anterior jugular vein; EJV: external jugular vein; IJV: internal jugular vein; SV: subclavian vein; SVC: superior vena cava.

The segmental anatomy of a fully developed AJVS is illustrated in the schematic drawing (Figure 4).

Schummer et al. [6] stated that correct placement of central venous catheters through the AJVS may be possible.

## 5. Literature Review

The literature covering collateral vein access for port insertion is rather limited. Teichgräber et al. [10] reported the placement of a port catheter through collateral veins in a patient with central venous occlusion through ipsilateral cervical collateral veins. Very few reports exist of the placement of a port through internal mammary veins [11, 12]. Walser [13] described and illustrated a case, in which a “port catheter was negotiated into the superior vena cava by way of a large, crossed-cervical venous collateral”, but without exact anatomical description of the veins and illustrating a catheter with probably just a subcutaneous “cross-cervical” route and then passing through a large mediastinal vein. Even intercostal veins may serve as transcollateral venous access for port placement; however, this procedure requires a thoracotomy [14]. To the best of our knowledge, the only published case roughly comparable to ours is the one of Marcy et al. [15]. Here, a description of a port implantation in a crossover technique, utilising puncture of a rather normal-sized left EJV, and subsequently through an anastomosis of the left and right AJV, naming it “anterior jugular arch anastomosis” and with greater enlargement of this than in our case, is presented. Marcy titled the procedure “external jugular vein cross-over.” In contrast to this, the access to the EJV was not an option in our patient, as her left EJV drained into the SV that in the further course was occluded and had no connection with the AJVS.

The important role of the AJVS as a collateral also becomes apparent in the context presented by Yamada et al. [16], where a case in which a malfunctioning implantable cardioverter-defibrillator lead was exchanged “via an angulated and tortuous collateral vein” was published. Here, lead placement is obviously via a crossover technique through the AJVS, accessed by subclavicular puncture of the occluded SV. Brieda et al. [17] reported on a patient, who was referred for upgrading of a dual-chamber implantable cardioverter-defibrillator to a biventricular cardioverter-defibrillator in the presence of a suboccluded left SV, “using a collateral vein that drained into the contralateral subclavian vein.” Again in this case, the collateral vein is not named. A look at the attached venogram clearly demonstrates an approach via a collateral vein of the SV and again a crossover technique via the AJVS.

## 6. Conclusion

We describe a case of placement of a port catheter by direct puncture of the horizontal lateral segment of the AJVS and crossover through the JVA that was technically possible without the use of special equipment, not necessitating greater effort or significantly higher costs than an ordinary port implantation, and feasible despite a tortuous and relatively narrow diameter JVA.

In the unusual constellation of patients with central venous occlusion on one side and requiring ipsilateral port implantation, closer consideration of a potentially fully developed and enlarged AJVS is warranted, as this vessel has been proven clinically to be a major cervical crossover collateral vein.

For clinicians, the AJVS can play an important role as a collateral for the insertion of port catheters, pacemaker leads, or other types of central devices.
